# Clinical multimodal Brillouin microscopy-optical coherence elastography system for lens biomechanics

**DOI:** 10.1117/1.JBO.30.12.124511

**Published:** 2025-12-12

**Authors:** Justin Schumacher, Christian Zevallos-Delgado, Leana Rohman, Alexander W. Schill, Manmohan Singh, Hongyuan Zhang, Marco Ruggeri, Jean-Marie Parel, Fabrice Manns, Kirill V. Larin, Giuliano Scarcelli

**Affiliations:** a University of Maryland, Department of Bioengineering, College Park, Maryland, United States; b University of Houston, Department of Biomedical Engineering, Houston, Texas, United States; c University of Miami, Department of Biomedical Engineering, Coral Gables, Florida, United States; d University of Miami Miller School of Medicine, Bascom Palmer Eye Institute, Ophthalmic Biophysics Center, Miami, Florida, United States; e Cole Eye Institute, Cleveland Clinic, Cleveland, Ohio, United States

**Keywords:** optics, Brillouin microscopy, optical coherence tomography, optical coherence elastography, presbyopia, ophthalmic instrumentation

## Abstract

**Significance:**

Estimating biomechanical properties of the *in vivo* crystalline lens remains a challenge and is a barrier to evaluating novel lens softening therapies. There is a need to estimate quantitative biomechanical properties of the human anterior and mid segments of the eye *in vivo* for conditions such as presbyopia.

**Aim:**

We aim to develop a multimodal elastography device that enables high-performance sequential 3D imaging with both Brillouin microscopy and optical coherence elastography (OCE).

**Approach:**

We combined Brillouin spectroscopy and OCE on a modified slit lamp platform for human measurements. The multimodal system was first characterized and then tested on both a porcine eye and a human subject.

**Results:**

Both OCE and Brillouin microscopy were characterized at peak operating performance for clinical imaging. Successful measurements of an *in situ* porcine lens and a human *in vivo* lens are reported.

**Conclusion:**

We demonstrated the first successful multimodal OCE and Brillouin microscopy measurement in a human subject. This instrument offers the potential to characterize the biomechanical status of presbyopia with age.

## 1 Introduction

Elastography techniques have flourished and become important tools in understanding biomechanics.^1^^–^^4^ Optical elastography techniques enable high-resolution mechanical mapping of tissues with high sensitivity while also maintaining a large field of view for examining larger structures. In particular, Brillouin microscopy and optical coherence elastography (OCE) have garnered attention because OCE has the capability of quantitative elastography by detecting nanometer-scale displacements in response to a controllable stimulus, and Brillouin microscopy can map mechanical properties with a noncontact confocal approach.^5^^–^^8^ Brillouin microscopy uses a phonon–photon interaction of light to probe thermally generated spontaneous acoustic waves in a material and measures the frequency shift of this interaction to derive mechanical properties of the material. Because this frequency shift is measured with optical spectroscopy in a confocal microscope, Brillouin microscopy has a high spatial resolution for mapping mechanical properties. In addition, Brillouin microscopy is a noncontact technique that is desirable for mechanical imaging because there is no mechanical perturbation of the sample. However, Brillouin microscopy assesses a high-frequency longitudinal modulus, which can only relate back to the quasi-static Young’s modulus with an empirically-derived log–log relationship that is specific to each material type.^9^^–^^11^ For more physiologically relevant Young’s modulus measurements, estimates can be made with wave-based OCE that relies on tracking mechanical waves propagating in tissues using optical coherence tomography (OCT) imaging.^12^ With OCE, a lower frequency mechanical wave is generated in the sample of interest, and the deformation is tracked across both space and time to measure a velocity that relates to the elastic modulus of the material. OCE can probe the Young’s modulus of a material directly but is limited to surface measurements in transparent tissues such as the crystalline lens due to limited scattering.^13^^,^^14^ In addition, the depth resolution of OCE is limited by the wavelength of the propagating surface shear wave. OCE and Brillouin microscopy have complementary strengths and weaknesses, so their combination is synergistic but optically challenging due to their different working principles.

The synergistic value of the two technologies for crystalline lens applications was recently demonstrated by side-by-side separate systems, leading to the successful calibration of the Brillouin modulus from OCE-measured Young’s modulus.^11^ Although the combination of OCT and Brillouin systems has been demonstrated recently, there have been no prior combinations of OCE and Brillouin. In earlier instruments for ophthalmic applications, only A-lines coaligned with the Brillouin system were collected for axial motion correction.^15^ Another device was developed for studying neural tube formation,^16^ where the acquisition of OCT B-mode images served as guidance for Brillouin acquisition. However, the system was not designed for either OCE imaging or human *in vivo* measurements. Previous maximum permissible exposure analysis and *in vivo* instruments have demonstrated the safe operation of Brillouin and OCE instruments for cornea and lens measurements.^8^^,^^15^^,^^17^^,^^18^ The combination of Brillouin and OCE modalities for successful *in vivo* measurements of the lens mechanical properties is challenging due to the anatomical position of the lens inside the eye, the need for precise co-alignment of the external force stimulus with the OCE and Brillouin imaging systems, and the presence of eye motion over time with acquisition.

In this work, we developed and characterized the first multimodal Brillouin microscopy-OCE device geared toward the clinic and demonstrated its application to study human *in vivo* lens mechanical properties.

## 2 Materials and Methods

### 2.1 Multimodal Instrument Design

The multimodal setup was first characterized and demonstrated on an *in situ* porcine lens imaged through the anterior segment of the eye and then used to measure a human subject. Measuring lens biomechanical properties is important to help better understand the mechanism of ocular accommodation, characterize the age-related stiffening of the lens leading to presbyopia, and assess the efficacy of presbyopia treatments that rely on lens softening.^19^

**Fig. 1:**
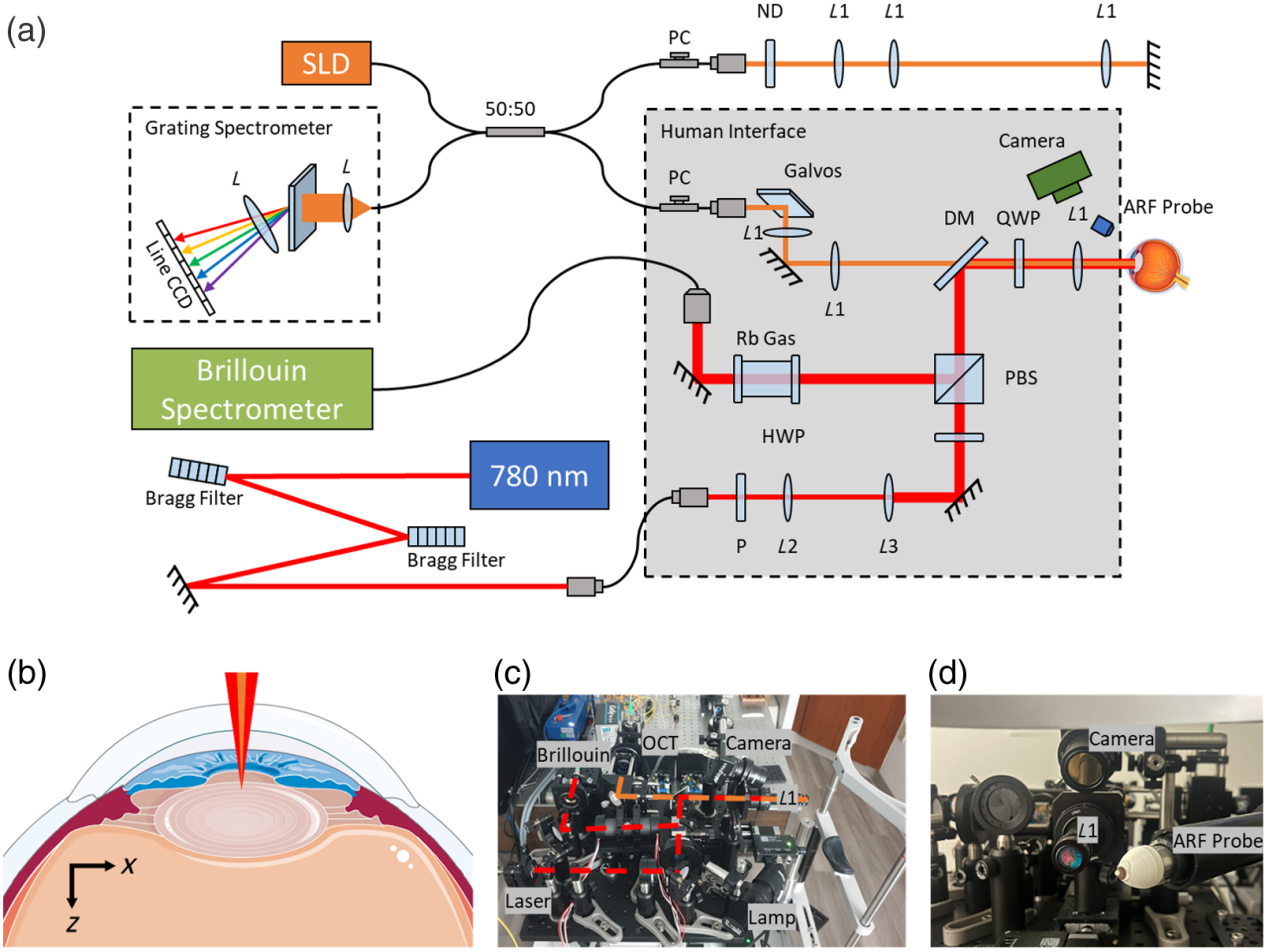
Combination of the Brillouin–OCE system. (a) The OCT system (orange) common arm and Brillouin confocal (red) sample arm were coaligned via a dichroic mirror on the human interface, which was a modified slit lamp for ophthalmic imaging. (b) Diagram of the lens imaging with scan axes labeled for OCE and Brillouin. (c) The human interface where the Brillouin–OCE system was implemented. (d) The subject’s view of the objective lens, lateral tracking camera, and acoustic radiation force stimulator. Eye image adapted from Servier Medical Art,^20^ licensed under CC BY 4.0.^21^

The combination Brillouin–OCE imaging system was built with a human interface mounted on a modified slit lamp table for clinical operation, as shown in Fig. 1. On the human interface, the OCE (orange beams) and Brillouin (red beams) imaging systems were coaligned on a common sample arm and objective lens, with laser sources and detectors using optical fibers to connect to the interface. Slit lamp positioning is aided visually with a small lateral tracking camera.

The Brillouin system uses a 780 nm CW tunable diode laser (DL Pro, TOPTICA Photonics, Gräfelfing, Germany), locked to a rubidium cell, and was spectrally cleaned with double Bragg gratings. The polarized laser beam was expanded with a telescope (L2=20  mm, L3=75  mm) and sent through a half-wave plate and polarizing beam splitter (PBS) to the objective. The objective was a 0.11 NA achromatic doublet (L1=50  mm) mounted on a scanning stage with a quarter-wave plate (QWP) to allow the backscattered Brillouin signal to return through the orthogonal PBS pathway. If desired, the back-reflected elastic background can be further cleaned with a heated rubidium cell before coupling into a single-mode fiber and sent to the spectrometer. The Brillouin spectrometer was based on a 15 GHz cross-axis double-VIPA spectrometer described previously^4^^,^^15^ and was chosen for its high speed and throughput when dealing with biological samples.

The OCE system was based on a spectral-domain OCT system combined with a spherically focused 3.5 MHz acoustic radiation force (ARF) transducer (V382-SU, Olympus Corp., Tokyo, Japan) to generate mechanical waves. Spectral domain OCT was chosen for its simplicity, relatively close wavelengths to the Brillouin source, and guaranteed displacement stability, which is important for OCE motion tracking. The light source was a superluminescent diode (SLD-mCS-371-HP1-SM, Superlum Diodes, Carrigtwohill, County Cork, Republic of Ireland) centered at 842 nm with a 50 nm bandwidth and 2.4 mW power on the sample. The OCT interferometer was Michelson-style, and the sample arm light was combined with the Brillouin light into the main objective via a steep long-pass dichroic mirror (DI03-R785-T3-25X36, IDEX Health & Science, LLC, Rohnert Park, California, United States). Traditionally, OCT B-mode imaging is achieved through a galvanometer-mounted mirror scanning from the back focal plane of the objective, but in the combination system, such an arrangement would put the galvanometer scanner too close to the objective and interfere with the placement of the DM and QWP. Therefore, a 4f imaging system was used to relay the scanning plane closer to the back focal plane of the objective lens. This compromised the OCT lateral scanning distance but was justified by simplifying the Brillouin signal return path. The lateral scanning distance was still large enough for an OCT signal to track a propagating mechanical wave, and polarization effects from the QWP were compensated with controllers on the return path to maximize the signal. To compensate for spectral dispersion, an equivalent 4f imaging system and objective lens (L1) were placed in the OCT reference arm. The OCT spectrometer (CS800-840/120-250-OC2K-CL, Wasatch Photonics, Morrisville, North Carolina, United States) operated at a 25 kHz A-line rate.

For OCE imaging, the ARF transducer was mounted and aligned to the focus of the OCT B-mode initial scan position to maximize the propagation distance across the scanning field of view. The ARF transducer distance was aligned using a custom 3D-printed cone filled with ultrasound coupling gel. The focal length of ∼21  mm was chosen to achieve easier focusing on the surface of the lens through the anterior chamber of the eye. The ARF transducer was driven with a waveform generator and RF amplifier and triggered synchronously with the OCT frame trigger in M-B-mode imaging.^7^

### 2.2 OCT and OCE System Characterization

**Fig. 2:**
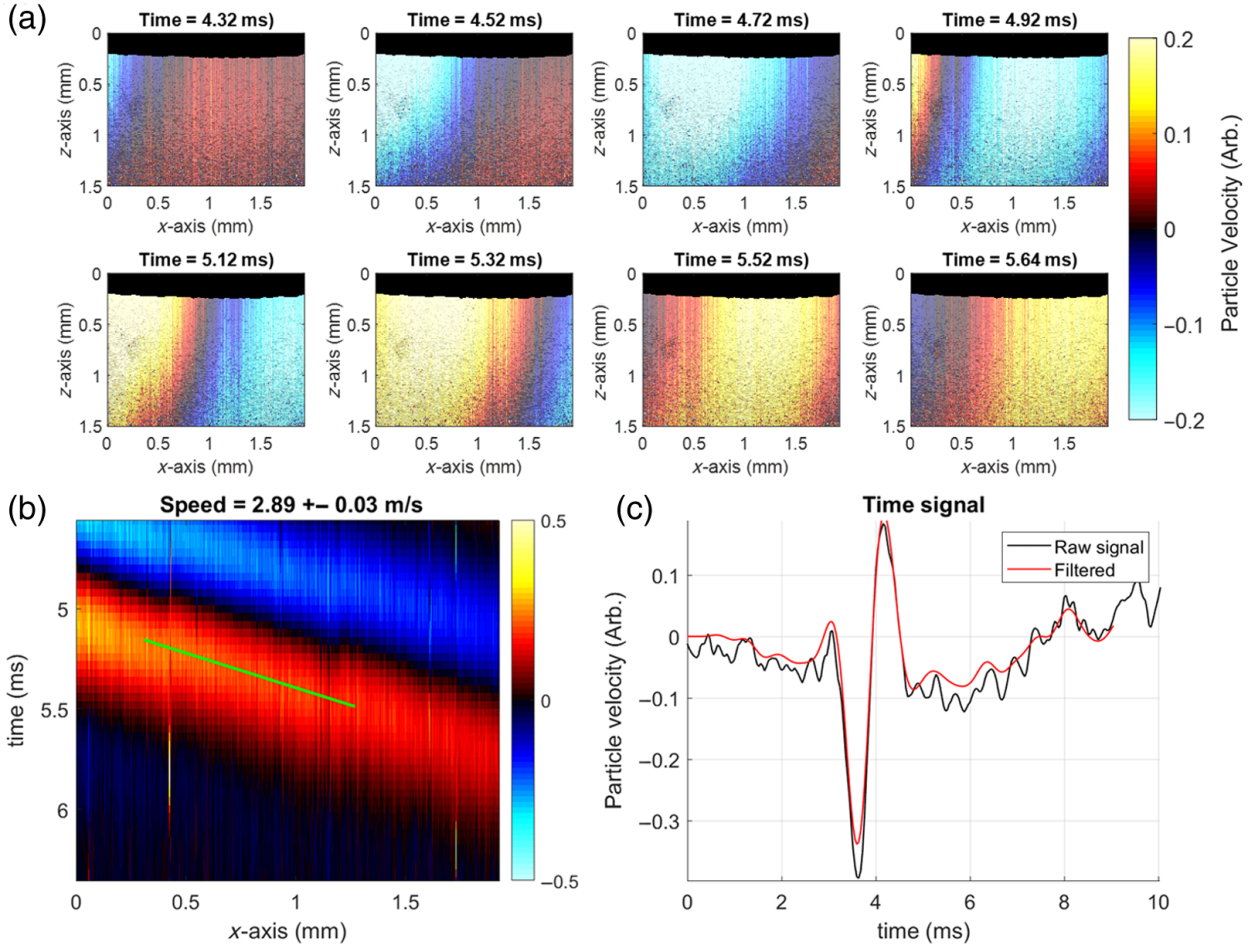
OCE system characterization of a 1% agar phantom with a single pulse. (a) The processed M-B-mode video frames and (b) the resulting space-time map. Color represents arbitrary particle velocity. (c) The deformation plot is shown before and after low-pass filtering.

Both the OCT and OCE systems were characterized in the multimodal Brillouin–OCE imaging system. For OCT imaging, the peak sensitivity of the system was measured at 98.7 dB with a sensitivity roll-off of −10.8  dB at a 2.2 mm imaging depth. The axial resolution was measured to be 9.21  μm, and the lateral resolution was measured to be 39.37  μm. These values agree with the theoretical estimates for resolution. The displacement stability of the OCT system was assessed in single (i.e., common-path) and dual arm implementations and was verified during alignment to have sub-nanometer stability on the optical table in the dual arm implementation. Initial OCE characterization on an agar phantom is shown in Fig. 2. A single pulse with a 500  μs width was used to probe the elastic wave group velocity in a 1% (w/w) agar phantom with 0.1% intralipid solution added as a scatterer. Once the ARF transducer was aligned to maximize the displacement signal, M-B-mode imaging^12^ was performed with a 20  μs exposure time per A-line and 1000 A-lines per M-mode scan. Representative video frames of the displacement are shown in Fig. 2(a). The data were processed according to existing methods,^22^ and the resulting space-time map of the surface propagation is shown in Fig. 2(b). The colors represent axial particle velocity, which is a change in deformation as the mechanical wave propagates through the sample. An example time series of the deformation after 800±600  Hz bandpass Butterworth filtering is shown in Fig. 2(c). Using this particle velocity as a marker of deformation, its change in position over time is fit to a line; its slope is the speed of the propagating wave. The group velocity was measured to be 2.89  m/s. This group velocity is in general agreement with wave-based OCE measurements in the literature for a similar type of 1% agar phantom.^7^

### 2.3 Brillouin System Characterization

**Fig. 3:**
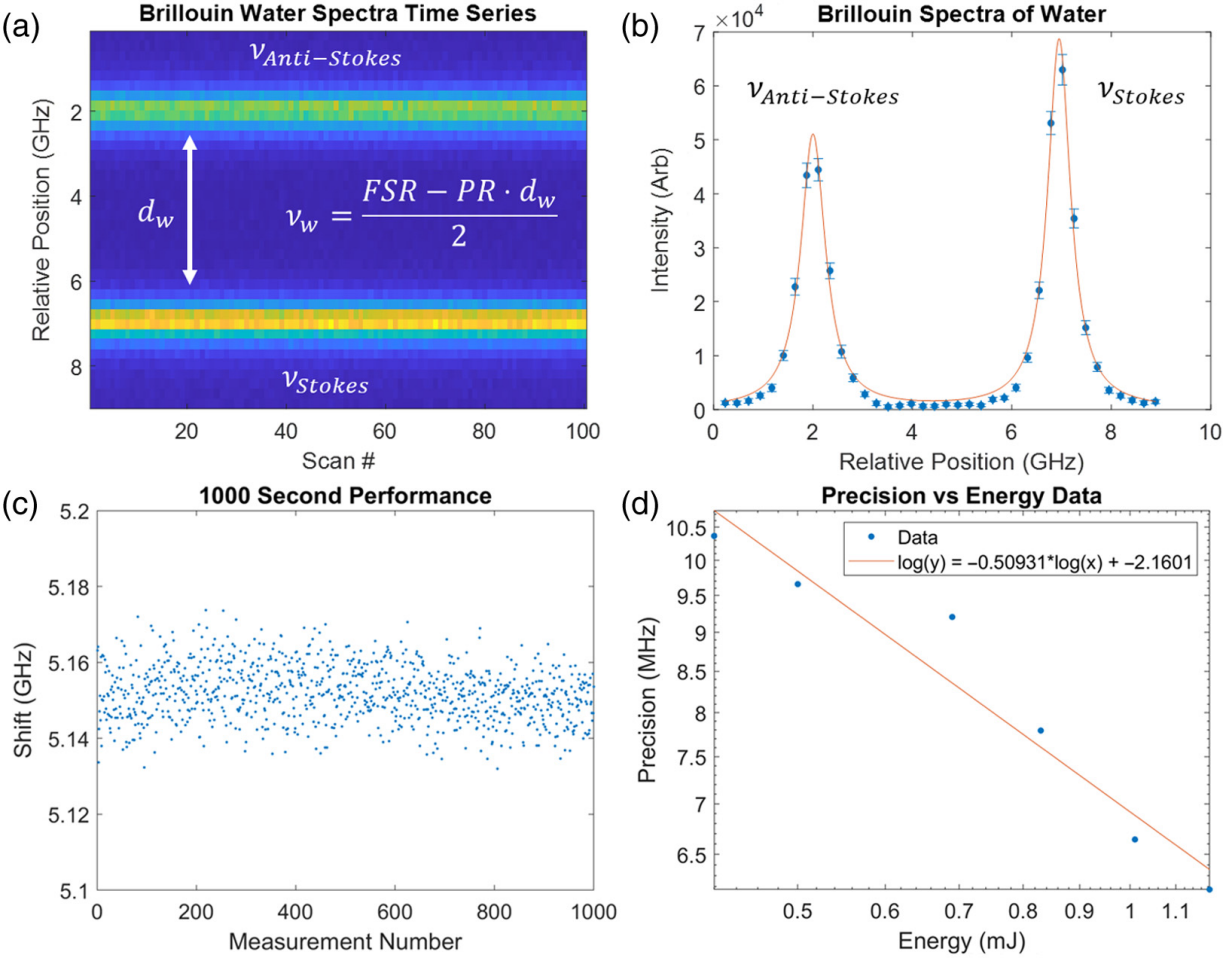
Brillouin system characterization in a water sample. Water was imaged in a quartz cuvette, and (a) the resulting Brillouin spectra were obtained and (b) fit with a Lorentzian curve. The vertical error bars are the standard deviation of the intensity, and the horizontal error bars are the shift precision from 100 samples. (c) Long-term scan precision was evaluated over 1000 s using water spectra. (d) The shift precision is plotted as a function of energy on the sample to determine the shot-noise-limited performance of the spectrometer.

The confocal Brillouin imaging system was characterized within the multimodal Brillouin–OCE imaging system. Unlike the OCE imaging system, where the galvanometer position deviates from ideal alignment, the Brillouin imaging system was prioritized for optimal alignment due to the low signal efficiency of the spontaneous Brillouin process. The performance of the Brillouin confocal imaging system is comparable to that of existing Brillouin imaging systems that use cross-axis VIPA spectrometers. Figure 3 shows the Brillouin spectroscopy characterization of the system. First, the system was calibrated with water and methanol standard samples with known Brillouin shifts. From this, the experimentally measured free spectral range (FSR) and GHz pixel ratio (PR) were reported as an FSR of 15.1 GHz and PR of 0.234  GHz/px consistent with a 15 GHz VIPA spectrometer design. Next, a quartz cuvette filled with water was measured, and the resulting Brillouin shifts were calculated based on previously published methods.^4^ Briefly, the cross-axis VIPA spectrometer contains the Stokes and Anti-Stokes Brillouin peaks from neighboring VIPA orders, and the distance between the peaks d relates to the Brillouin shift $\nu_{B}$ from the relationship: $\mathrm{FSR}=2\cdot \nu_{b}+PR\cdot d$. The resulting calibration profile of water is shown in Figs. 3(a) and 3(b). The long-term precision of the system was evaluated by measuring the Brillouin water spectra every second for 1000 s, and the resulting data are shown in Fig. 3(c). The long-term precision was reported as 7.21 MHz standard deviation. The shot-noise-limited operation of the system was verified by estimating the precision of the Brillouin shift of water as a function of incident energy. The resulting curves shown in Fig. 3(d) confirm both shot-noise-limited operation and acceptable precision at safe laser energies in *in vivo* measurements.

### 2.4 Porcine Sample Preparation and *In Situ* Imaging

We demonstrated successful *in situ* imaging of a porcine lens through the intact cornea sequentially with both OCE and Brillouin systems. A fresh eye was acquired on the day of the experiment from a local butcher (Wagner Meats, Mt Airy, Maryland, United States) and placed on ice until measurements began. The eye was brought to room temperature before scanning and assessed visually for a red reflex to confirm the absence of cold cataracts. Once the eye was checked for quality, both OCE and Brillouin imaging were acquired sequentially. For OCE imaging, the ARF transducer focal point was positioned at the periphery of the OCT imaging field of view to allow for maximum wave propagation imaging. For *in situ* imaging, five cycles of 1 kHz pulses were used to probe the group wave speed across the lens surface. For Brillouin imaging, the ARF transducer was pulled away from the eye, and an axial scan of 10 mm was taken along the approximate center of the OCT image. To help correct optical aberrations due to the porcine cornea surface, a glass coverslip was placed at the apex of the cornea after topical instillation of methylcellulose drops.

### 2.5 Human Subject *In Vivo* Imaging

We demonstrated the first successful multimodal OCE and Brillouin human measurements. The human subject provided informed consent in accordance with the tenets of the Declaration of Helsinki and an approved protocol by the University of Miami’s institutional review board. One eye of a healthy 54YO man was measured sequentially using a similar protocol to the porcine measurements but lowering the Brillouin laser power to 7 mW and ARF power based on previously established safety limits.^18^ Brillouin and OCE measurements were acquired sequentially. After the acquisition of the Brillouin image, a topical anesthetic was applied to the ocular surface, and ultrasound coupling gel was applied to the ARF probe. Once the OCE imaging system was aligned, the ARF probe was slowly moved forward until the gel was in contact with the eye near the limbus. The OCE measurement was then acquired. Both Brillouin and OCE images required motion correction in depth, with OCE phase displacements requiring additional broad median filtering to remove excess motion.

## 3 Results

### 3.1 Porcine *In Situ* Lens Measurements

**Fig. 4:**
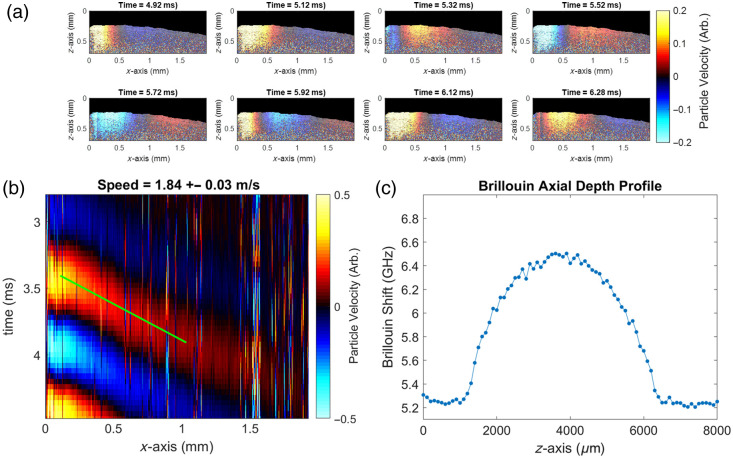
Exemplary multimodal Brillouin–OCE measurements taken on the same *in situ* pig lens. (a) The processed M-B-mode video frames of the elastic wave propagation in the lens. (b) The OCE elastic wave speed maps across the surface of the lens. Color represents arbitrary particle velocity. (c) The Brillouin shift depth profile was measured along the optical z-axis. Note how the intensity of the colormap for OCE group speed is reliable for the first 400  μm along the optical z-axis, whereas the Brillouin shifts are measured along the entire thickness of the lens.

A porcine eye was imaged sequentially with the multimodal system, as shown in Fig. 4. Representative video frames showing a portion of the propagation are shown in Fig. 4(a). The resulting OCE-derived space-time map is shown in Fig. 4(b). The group velocity of the lens after filtering with a 200 Hz bandpass Butterworth filter centered at 1 kHz was measured to be 1.84  m/s. This group velocity was higher than previously reported values for air-puff OCE,^11^ likely due to different stimulation and imaging conditions between these instruments, e.g., air-pulse and *ex vivo* versus ARF and *in situ*. The resulting Brillouin depth profile is shown in Fig. 4(c).

### 3.2 Human *In Vivo* Lens Measurements

**Fig. 5:**
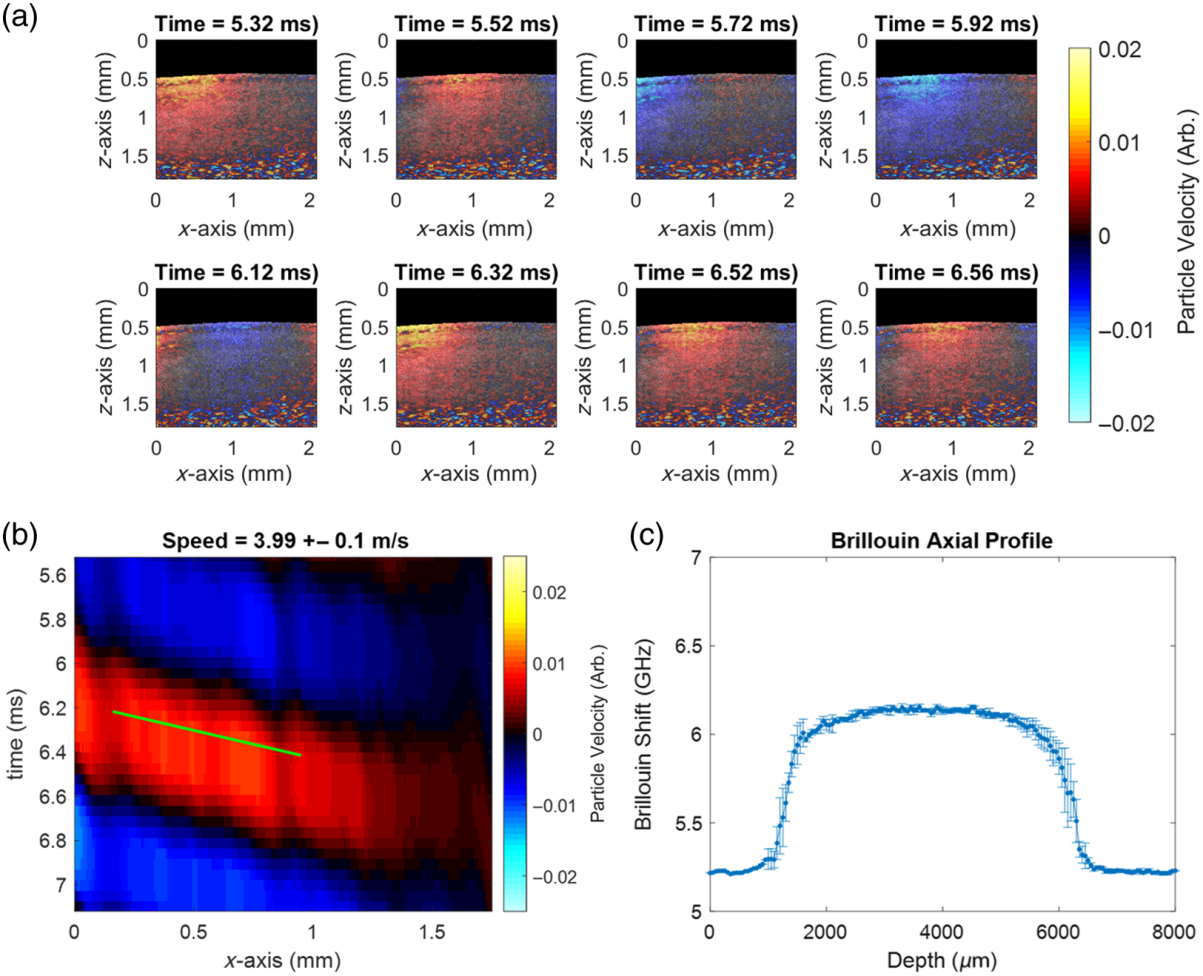
Exemplary multimodal Brillouin–OCE measurements taken on the same human *in vivo* subject. (a) The processed M-B-mode video frames of the elastic wave propagation. Color represents arbitrary particle velocity. (b) The OCE filtered space-time map measured across the surface of the lens. (c) The Brillouin shift depth profile measured along the optical z-axis from three exemplary traces.

The results of sequential multimodal imaging in a human subject are shown in Fig. 5. There is noticeable wave propagation shown in the representative video frames in Fig. 5(a). The OCE-derived space-time map is shown in Fig. 5(b) along the surface of the lens, and the group velocity of the lens was measured to be 3.99  m/s. The Brillouin shift profiles in Fig. 5(c) are expected for an older human lens when compared with previous work.^23^

## 4 Discussion and Conclusion

In this work, we have demonstrated the feasibility of the multimodal OCE-Brillouin imaging system, as well as the performance characterization of both the OCT, OCE, and Brillouin sub-systems. The spectral-domain OCT system operated at high sensitivity and achieved significantly shortened exposure times compared with those reported for an existing Brillouin-OCT setup.^15^ This translates into better wave tracking at the surface of the lens when the OCE system is in operation.

In this OCT setup, the lateral scanning range was around 2 mm due to galvanometer placement, which currently limits wave tracking across the entire lens surface. This tradeoff was chosen to avoid placement of the scanning system into the common path, which would reduce the already weaker Brillouin intensity compared with the OCT signal. Despite the shortened lateral scanning range, the OCE system was still capable of tracking elastic wave propagation in M-B-mode operation in Fig. 2, and the ability to track waves along the lens surface has not been affected, as shown in Fig. 4.

Brillouin spectroscopy has been well-defined for *in vivo* operation, and the results of these measurements confirm successful characterization in Fig. 3 and operation in the lens in Fig. 4(c). Although the high system extinction granted by the Rubidium cell is useful for highly reflective samples where extinction requirements may be higher, such as the cornea, the lens is well placed inside the eye where air–tissue reflections are minimal. Although other cross-axis VIPA spectrometers use techniques to improve extinction in biological samples, such as apodization and coronagraphy,^4^ the high extinction achieved in this Brillouin spectrometer for lens applications does not require these techniques. Instead, the system was tuned to maximize the collection efficiency of the backscattered Brillouin light. One potential system limitation is that in the crystalline lens, the cross-axis VIPA spectrometer may lower the ability to detect mechanical heterogeneity, e.g., interfaces at the lens nucleus where the Brillouin width is experimentally wider. More data will need to be measured to assess the importance of this observation *in vivo*.

The porcine data in Fig. 4 show successful *in situ* imaging of both a mechanical averaging of the elastic modulus at the surface of the lens in Fig. 4(b), as well as a high-resolution spatially varying Brillouin shift throughout the lens in Fig. 4(c). Based on the OCE-measured wave speed of 1.84  m/s and known 1 kHz excitation frequency, a wavelength can be estimated as 1.84 mm, which represents the sensing depth of the propagating shear wave that contributes to the elastic modulus estimation. It is clear from the OCE wave propagation timeframes that the current OCT-based tracking cannot measure scattering deep enough in the lens to resolve the full wavelength depth but is still capable of making a measurement of the shear wave speed from the surface propagation. By comparing this wavelength with Brillouin depth scans, there is a spatially varying modulus based on Brillouin measurements that is contributing to the mechanical averaging of the elastic modulus as measured by OCE.

The human subject measurements in Fig. 5 show successful *in vivo* imaging of a human subject with both OCE and Brillouin co-registered on a single multimodal instrument. The imaging shows both a surface-level mechanical averaging of the elastic modulus represented by the propagating wave speed, as well as a spatially varying longitudinal modulus represented by the Brillouin shift. This corresponds to a wavelength estimated as 3.99 mm, which is a significant component of the lens, including both cortex and nucleus layers. Taken together, this implies that OCE-derived measurements are sensitive to spatially varying changes in both cortex and nucleus as well as their spatial amounts. Previous work has looked at the relationship between these quantities in porcine lenses using gold standard compression testing.^24^ With multimodal OCE and Brillouin systems, future studies will be able to understand this relationship *in vivo*, which is of great clinical interest.

With the current implementation, OCE measurements were taken along the lateral surface of the lens, and Brillouin measurements were taken along the depth axis of the lens. However, the current implementation of the Brillouin–OCE system allows for 3D imaging of both modalities. Future measurements can explore different mapping strategies, such as axial and lateral scanning of the Brillouin confocal microscope. In addition, OCE measurements can be optimized in frequency selection for the ARF excitation pulses, which may help sample different wavelengths in depth that contribute to mechanical averaging. Finally, both OCE and Brillouin imaging techniques can make estimates of attenuation, and this would relate to viscosity mapping of a sample. Although not included in our current system characterization, this remains a complex and interesting topic of future research.

In conclusion, we have developed a multimodal Brillouin–OCE imaging system developed to perform *in vivo* biomechanical measurements of the human crystalline lens. The system was characterized and tested on a live human subject. This is the first system of its kind capable of performing human *in vivo* OCE and Brillouin measurements on a single instrument. This instrument promises to be a unique tool for understanding lens biomechanics *in vivo* and assessing the efficacy of novel anti-presbyopia lens softening therapies.

## Data Availability

Data underlying the results presented in this paper are available upon request.
